# miR-92a-1-5p enriched prostate cancer extracellular vesicles regulate osteoclast function via MAPK1 and FoxO1

**DOI:** 10.1186/s13046-023-02685-2

**Published:** 2023-05-02

**Authors:** Lijuan Yu, Bingdong Sui, Xin Zhang, Jiayun Liu, Xiaoke Hao, Lei Zheng

**Affiliations:** 1 Department of Laboratory Medicine, Nanfang Hospital, Southern Medical University, Guangzhou, 510515 China; 2 Institute of Laboratory Medicine Center of Chinese People’s Liberation Army (PLA), Xijing Hospital, Fourth Military Medical University (Air Force Medical University), , Xi’an, 710032 China; 3 Krefting Research Centre, Institute of Medicine, University of Gothenburg, 410530 Gothenburg, Sweden; 4 Research and Development Center for Tissue Engineering, School of Stomatology, Fourth Military Medical University (Air Force Medical University), Xi’an, 710032 China; 5 College of Medicine, Northwest University, Xi’an, 710032 China

**Keywords:** miR-92a-1-5p, miR-92a-1-5p enriched EVs, Bone diseases, Bone metabolism, Osteoclast differentiation

## Abstract

**Background:**

We have previously reported that extracellular vesicles (EVs) derived from osteoblastic, osteoclastic and mixed prostate cancer cells promote osteoclast differentiation and inhibit osteoblast differentiation via transferring miR-92a-1-5p. In the present study, we focused on engineering miR-92a-1-5p into EVs and determining any therapeutic roles and mechanisms of the engineered EVs.

**Methods:**

A stable prostate cancer cell line (MDA PCa 2b) overexpressing miR-92a-1-5p was constructed by lentivirus, and EVs were isolated by ultracentrifugation. The overexpression of miR-92a-1-5p in both cells and EVs was tested using qPCR. Osteoclast function was evaluated by Trap staining, mRNA expression of osteoclastic markers *ctsk* and *trap*, immunolabeling of CTSK and TRAP and microCT using either in vitro and in vivo assays. Target gene of miR-92a-1-5p was proved by a dual-luciferase reporter assay system. siRNAs were designed and used for transient expression in order to determine the role of downstream genes on osteoclast differentiation.

**Results:**

Stable overexpression cells of miRNA-92a-5p was associated with EVs upregulating this microRNA, as confirmed by qPCR. Further, miR-92a-1-5p enriched EVs promote osteoclast differentiation in vitro by reducing MAPK1 and FoxO1 expression, associated with increased osteoclast function as shown by TRAP staining and mRNA expression of osteoclast functional genes. siRNA targeting MAPK1 or FoxO1 resulted in similar increase in osteoclast function. In vivo, the miR-92a-1-5p enriched EVs given via i.v. injection promote osteolysis, which was associated with reduction of MAPK1 and FoxO1 expression in bone marrow.

**Conclusion:**

These experiments suggest that miR-92a-1-5p enriched EVs regulate osteoclast function via reduction of MAPK1 and FoxO1.

**Graphical Abstract:**

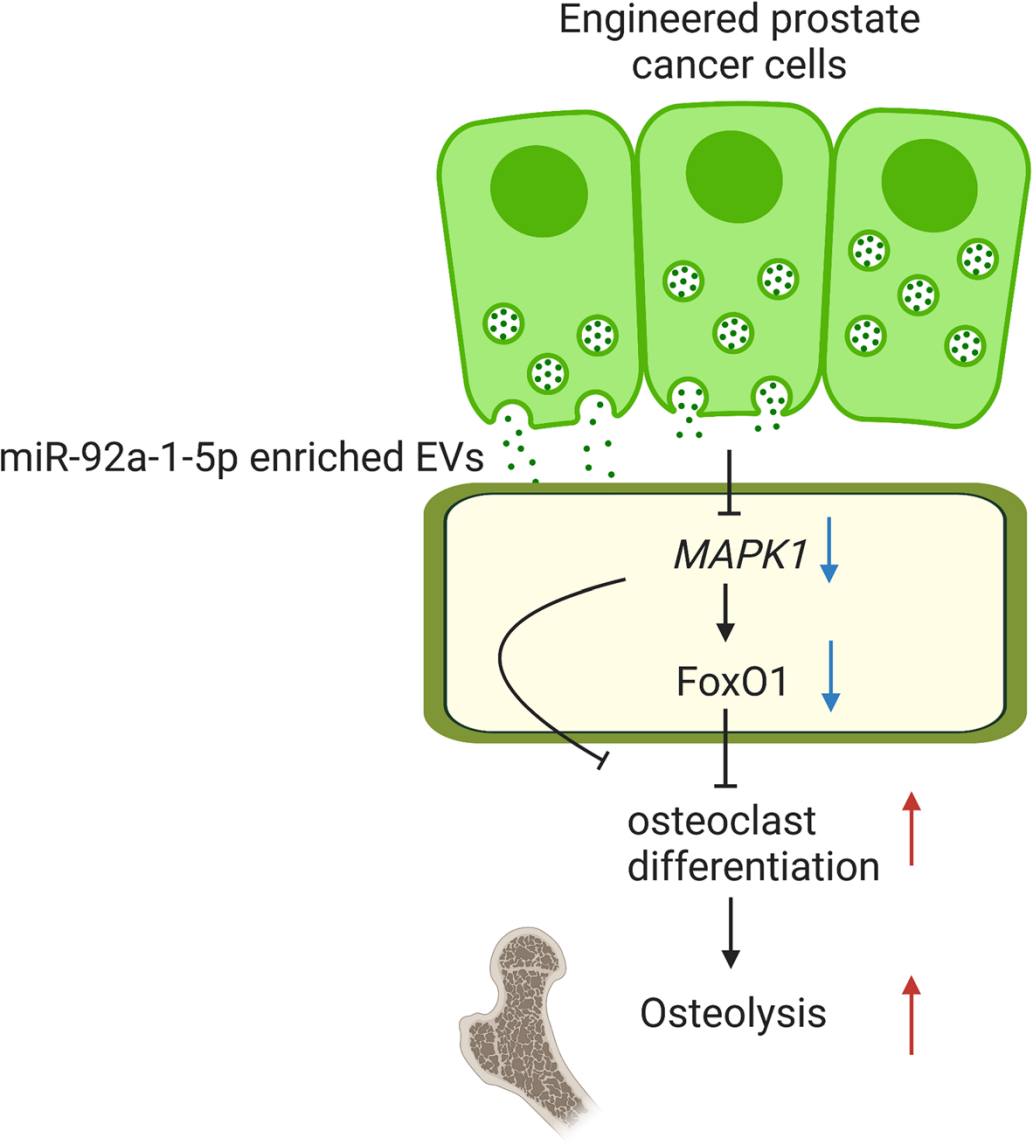

**Supplementary Information:**

The online version contains supplementary material available at 10.1186/s13046-023-02685-2.

## Background

Extracellular vesicles (EVs) and their cargoes are widely considered to mediate intercellular and inter-organ communication [1-4]. Among these cargoes, microRNAs (miRNAs) have been studied most thoroughly and have been demonstrated to be critical regulator of multiple diseases [5, 6]. From their biogenesis and composition, single miRNAs and miRNAs encapsuled in EVs are entirely different. Evidences support that miRNAs encapsuled in EVs are selectively sorted into EVs through a purposeful rather than passive process, with the help of RNA-binding proteins [7]. And, subsequently, miRNA-RNA binding protein complex is selectively degraded within multivesicular bodies (MVBs) [8]. Recently, breakthrough progress has reported that multiple motifs that contribute to sEV sorting and cellular retention in a cell-type-specific manner [9]. Meanwhile, miRNAs encapsuled in EVs are protected by phospholipid bilayer, which makes them more stable than single miRNAs and more suitable as therapeutic targets. However, the method to engineering EVs with potential therapeutic miRNA are still unclear.

We have previously reported that extracellular vesicles (EVs) derived from osteoblastic (MDA PCa 2b), osteoclastic (PC3) and mixed (C4-2) prostate cancer (PCa) cells promote osteoclast differentiation and inhibit osteoblast differentiation [10]. Furthermore, one of the miRNA cargoes, miR-92a-1-5p, was preliminarily identified as an important factor that promote osteoclast differentiation and meanwhile inhibit osteoblastogenesis [10]. Based on this, in the present study, we focused on engineering PCa EVs with this potential therapeutic miRNA. Nowadays, approaches used for enrichment/loading miRNA into EVs are accomplished by isolating EVs from cell line or body fluids and then loading them with the targeted miRNA by using electroporation and sonication [11]. However, contamination of reagent [12], aggregation of EVs [13], aggregation of miRNA [13], and leakage of endogenous cargo due to pore formation in EVs during the process have been drawing increasing attention from the EVs drug delivery community. Here, we proposed an assay to produce EVs carrying the desired therapeutic miRNA as a valid alternative to the previously described methods.

To generate miR-92a-1-5p engineered EVs we employed lentivirus-mediated overexpression system to establish miR-92a-1-5p stable overexpressed cell line. The overexpression of miR-92a-1-5p was verified by qPCR in both cells and EVs. We further aimed to investigate the roles and cellular mechanisms of action of the engineered EVs in osteoclast functions. Several in vitro and in vivo assays, were performed to assess the osteoclast function after coculture with miR-92a-1-5p enriched EVs. Specifically, a mechanism through which the engineered EVs produce their effect was identified. This enhanced understanding of the method to construct therapeutic miRNA enriched EVs and the roles and mechanisms of miR-92a-1-5p enriched EVs may contribute to the development of therapeutic nano-tools for osteoblastic bone diseases.

## Materials and methods

### Cell culture

The PCa cell line MDA PCa 2b was purchased from American Type Culture Collection (ATCC, USA). Raw264.7 cells were a kind gift from Dr. Weihua Yu at Air Force Medical University, China. MDA PCa 2b cells were cultured in F12K medium (ATCC) containing 20% fetal bovine serum (FBS). Raw264.7 cells were cultured in H-DMEM (Gibco, USA) containing 10% FBS. 100 μg/mL streptomycin and 100 U/mL penicillin (HyClone, USA) were supplemented. BMMs were prepared and cultured according to previously reported protocols [10].

### Lentivirus transfection

Lentivirus (pHB-U6-mir-92a-1-5p-EF1-LUC-PURO) were constructed at HANBIO (Shanghai, China). Lentivirus transfection was performed as instructions from the producer. The multiplicity of infection (MOI) for MDA PCa 2b was 10, and transfected cells were selected using 4 μg/mL puromycin for 6 days and then transferred to a medium lacking puromycin. MDA PCa 2b cells stably overexpressing miR-92a-1-5p were selected with puromycin for 4 days every 30 days.

### EVs isolation from cell culture medium

EVs were isolated using differential ultracentrifugation as reported [10]. Briefly, 210 mL of culture supernatant was harvested from 90%-confluent cells cultured for 48 h with 10% exosome-depleted FBS (VivaCell Biosciences, China) and centrifuged at 300 × *g* for 10 min, 2000 × *g* for 10 min, and then 10,000 × *g* for 30 min. Lastly, the supernatant or resuspension was centrifuged (Type 70 Ti Rotor, Beckman Coulter, USA) twice at 120,000 × *g* for 70 min, and the final pellet was resuspended in 200 μL of PBS.

### EVs characterization

EVs yield was measured using a BCA Protein Assay Kit (Pierce™, Thermo Fisher Scientific, USA), and then exosome morphology was analyzed using negative staining and TEM (Tecnai, USA), as described [14]. NTA was performed with a ZetaView instrument (Particle Metrix, Germany) at Echo Biotech Company.

### Western blotting

Western blotting of EVs (10 μg) or cell-lysate proteins (30–50 μg) was performed as previously described (with minor modifications) [10]. The primary antibodies (1:1000; Cell Signaling Technology, USA or Abcam, USA) were against the following targets: CD9, Alix, Annexin V, GM130, GAPDH, MAPK2/MAPK1, pMAPK2/MAPK1, FoxO1, TRAP and CTSK.

### Uptake of EVs by recipient cells

The EVs (10 μg) were labeled with Vybrant DID (Life Technology), incubated at 37 ℃ for 20 min, and then centrifuged at 120,000 × g for 90 min to remove the excess dye. The recipient cells were incubated with the labeled EVs in the dark for 90 min, washed once with PBS, fixed with 4% paraformaldehyde (PFA) for 10 min, and then stained with DAPI and fluorophore-conjugated phalloidin to visualize the nucleus and the (F-actin) cytoskeleton, respectively.

### EVs/cellular total RNA purification and amplification

Total EVs RNA was extracted with a Total Exosome RNA and Protein Isolation Kit (Life Technology, USA). EVs miRNA expression was normalized by cel-miR-39 as a spike-in control as recommended by the manufacturer. Total cellular RNA was extracted with the miRNeasy Mini Kit (QIAGEN, Germany). U6 was employed as the internal control for cellular miRNA. For qPCR, the Mir-X™ miRNA First Strand Synthesis kit (Takara, Japan), PrimeScript™ RT Master Mix kit (Takara), and SYBR® Premix Ex Taq™ II (Takara) were used. The miRNA qPCR primer sets were purchased from RiboBio and mRNA qPCR primers were synthesized by Tsingke (Beijing, China). The qPCR primers used were listed in Supplementary Table S1.

### In vitro osteoclast induction

Osteoclasts were induced as previously described [10]. Briefly, Raw264.7 cells were inoculated into 96-well plates at a density of 1 × 10^4^ cells/well, co-cultured with exosomes or siRNAs, and then cultured in the presence or absence of RANKL (100 ng/mL; Peprotech, USA). Trap staining (Sigma-Aldrich, USA), Trap-activity detection (Beyotime, China) and osteoclastic markers mRNA expression were employed to detect the osteoclast induction.

### Target-gene prediction

Targetscan, miRanda, and miRDB were used for miRNA target-gene prediction. We used the tool DIANA to discover the pathways enriched by the miRNA targets (http://www.microrna.gr/miRPathv3) [15], and then used David 6.8 Bioinformatics Resources (https://david.ncifcrf.gov/) to analyze the functional annotations [16].

### Luciferase reporter assay

Raw264.7 cells were co-transfected with miRNA and luciferase vectors containing wild-type or mutant 3′-UTR of MAPK1 with Lipofectamine 3000 (Invitrogen, USA). A Dual-Luciferase Reporter Assay System (Beyotime) was used for luciferase activity detection at 48 h post-transfection.

### Animal studies

The animal work was approved by the Animal Ethic Committee at Air Force Medical University, China. Male BALB/C nude mice were purchased from SJA Laboratory Animal Co. (Changsha, China). The mice were assigned in random order and accommodated to the facility for one week prior to the start of the experiment.

### Mice bone marrow education

Mice bone marrow education were administered by tail vein injection. As previously reported [10], mice (7 weeks old, *n* = 5 per group) were injected with 10 μg EVs (in 100 μL of PBS) into the tail vein thrice weekly for 4 weeks. PBS was used in the control group. On day 28, mice were sacrificed, and specimens were collected for different experiments.

### Micro-CT analysis

Micro-CT analysis was performed as previously described [17, 18]. Briefly, tibia sections were harvested and post-fixed in paraformaldehyde. Subsequently, 7 mm blocks were prepared, and 120 min micro-CT scan was used to image the specimen with a voltage of 80 kV, a current of 80 μA, and a resolution of 8 μm.

### Bone immunofluorescence assay

Mice’s left tibia were isolated, fixed in 4% PFA, quickly decalcified, and embedded in paraffin wax; subsequently, specimens were sectioned at 3-μm thickness by using a tissue slicer (Leica, Germany) away from light. Nuclei and target markers in the bone marrow were stained with DAPI (blue) and anti-MAPK1/FoxO1, respectively. Samples were imaged using a Nikon A1R confocal microscope (Nikon, Japan) or scanned using a case viewer system (3DHISTEC, Hungary).

### Statistical analysis

Statistical analysis was performed using GraphPad Prism version 7.0. Comparisons were performed using *t* tests and one-way ANOVA, as appropriate, *P* < 0.05 was considered statistically significant.

## Results

### Generation of miR-92a-1-5p enriched EVs

**Fig. 1:**
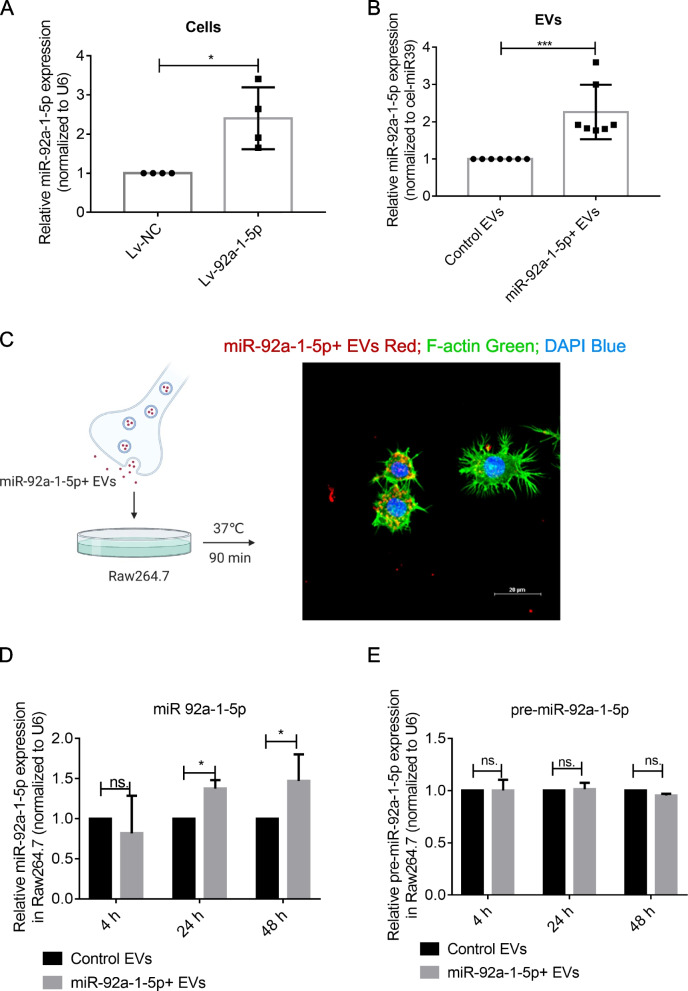
miR-92a-1-5p enriched EVs are successfully constructed. **A** Upregulation of miR-92a-1-5p in MDA PCa 2b cells transfected with miR-92a-1-5p-overexpressing lentivirus. **B** miR-92a-1-5p upregulation in EVs derived from MDA PCa 2b cells stably overexpressing miR-92a-1-5p. **C** Schematic representation of the designed experiment. Confocal microscopy revealed miR-92a-1-5p enriched EVs internalization by Raw264.7 cells after 90 min of co-culture at 37 °C. Scale bar = 20 μm. **D-E** qPCR analysis of mature miR-92a-1-5p and pre-miR-92a-1-5p in Raw264.7 cells at different time points after treatment with 10 μg EVs. Data were analyzed using *t* test. *, *P* < 0.05; ***, *P* < 0.001

miR-92a-1-5p was stably overexpressed in MDA PCa 2b cells by lentiviral vector (pHBLV-U6-miR-92a-1-5p-EF1-fLUC-T2A-Puro) transfection (Figure S1). miR-92a-1-5p overexpression was confirmed by qPCR with an approximately two fold up (Fig. 1A). In order to collect miR-92a-1-5p enriched EVs, stably expression cells were cultured in depleted medium for 48 h. Total RNA from EVs revealed a miR-92a-1-5p increment of two fold up (Fig. 1B).

Next, we characterized control EVs and miR-92a-1-5p enriched EVs (miR-92a-1-5p + EVs). Transmission electron microscopy (TEM) analyses revealed that single vesicles within the isolated vesicle mixtures showed an ovoid morphology, and no difference was observed among the groups (Fig S2A). Furthermore, the results of nanoparticle tracking analysis (NTA) demonstrated that the major size-distribution peaks of all three groups were in the 90–130 nm range (Fig S2B). Comparison between NTA results and TEM confirmed that these vesicles were not any other particles of similar size, as previously reported [11]. The presence of EVs markers (CD9, CD63, CD81, Alix, and Annexin V) by western blotting and flow cytometry analyses (Fig S2C-D) reinforced the quality of the isolated EVs. In add, the Golgi marker GM130 was not detected in the isolated EVs (Fig S2C). We also discovered the EVs yield of the three groups, and we didn’t find the significant difference of EVs yield between control EVs and miR-92a-1-5p + EVs.

Next, miR-92a-1-5p + EVs were added to the medium of Raw264.7 cells, and their rapid internalization were observed within 90 min after co-culture (Fig. 1C). In this condition, the expression of mature miR-92a-1-5p increased at 24 h and 48 h (Fig. 1D), whereas the level of pre-miR-92a-1-5p was unaltered (Fig. 1E). These results suggested that the increased expression was due to the transfer of mature miR-92a-1-5p through miR-92a-1-5p + EVs.

### miR-92a-1-5p enriched EVs promotes osteoclast differentiation

Our aforementioned study reported that prostate cancer EVs can promote osteoclast differentiation and attenuate osteoblast differentiation, both at least partly mediated by transferring miR-92a-1-5p [10]. Here, we intended to investigate the role of miR-92a-1-5p + EVs in bone homeostasis.

**Fig. 2:**
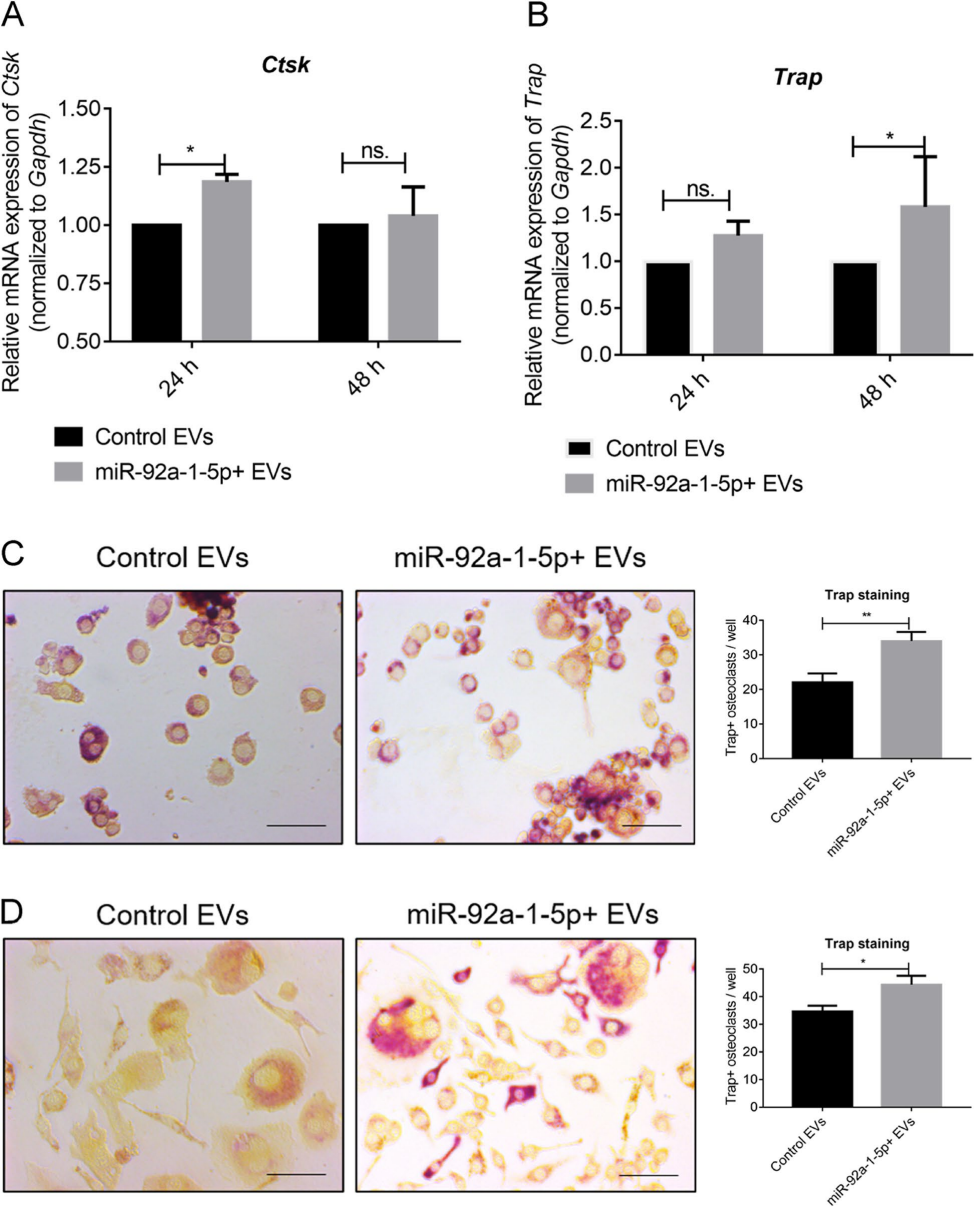
miR-92a-1-5p enriched EVs promotes osteoclast differentiation. **A-B** *Ctsk* and *Trap* expression was determined using qPCR at 24 and 48 h after treatment with 20 μg/mL EVs in normal culture medium. **C** Trap + osteoclasts were identified and quantified after treatment for 6 days in Raw264.7 cells with EVs in normal medium. Scale bar = 50 μm. **D** Trap + osteoclasts were identified and quantified in BMMs after treatment for 6 days with EVs in normal medium. Scale bar = 50 μm. Data were analyzed using *t* test. *, *P* < 0.05; **, *P* < 0.01

Raw264.7 cells were incubated with control EVs or miR-92a-1-5p + EVs for 24 and 48 h in the absence of RANKL and then subject to qPCR analysis, which revealed that miR-92a-1-5p + EVs markedly induced the mRNA expression of *Ctsk* and *Trap* in the cells (Fig. 2A-B). Further, in the absence of RANKL, osteoclast differentiation was markedly promoted by miR-92a-1-5p + EVs in both Raw264.7 cells and bone marrow macrophages (BMMs) by Trap staining after 6 days coculture (Fig. 2C-D). Next, we also detected the protein expression of CTSK and TRAP by western blotting after 48 h co-culture of EVs in Raw264.7 cells. CTSK and TRAP expression were both upregulated after 48 h (Figure S3). We previously reported that osteoclast differentiation can be induced by PCa EVs without RANKL [10]; here, we shown that miR-92a-1-5p + EVs can strengthen this effect with or without RANKL.

### MAPK1 is the direct target for miR-92a-1-5p

**Fig. 3:**
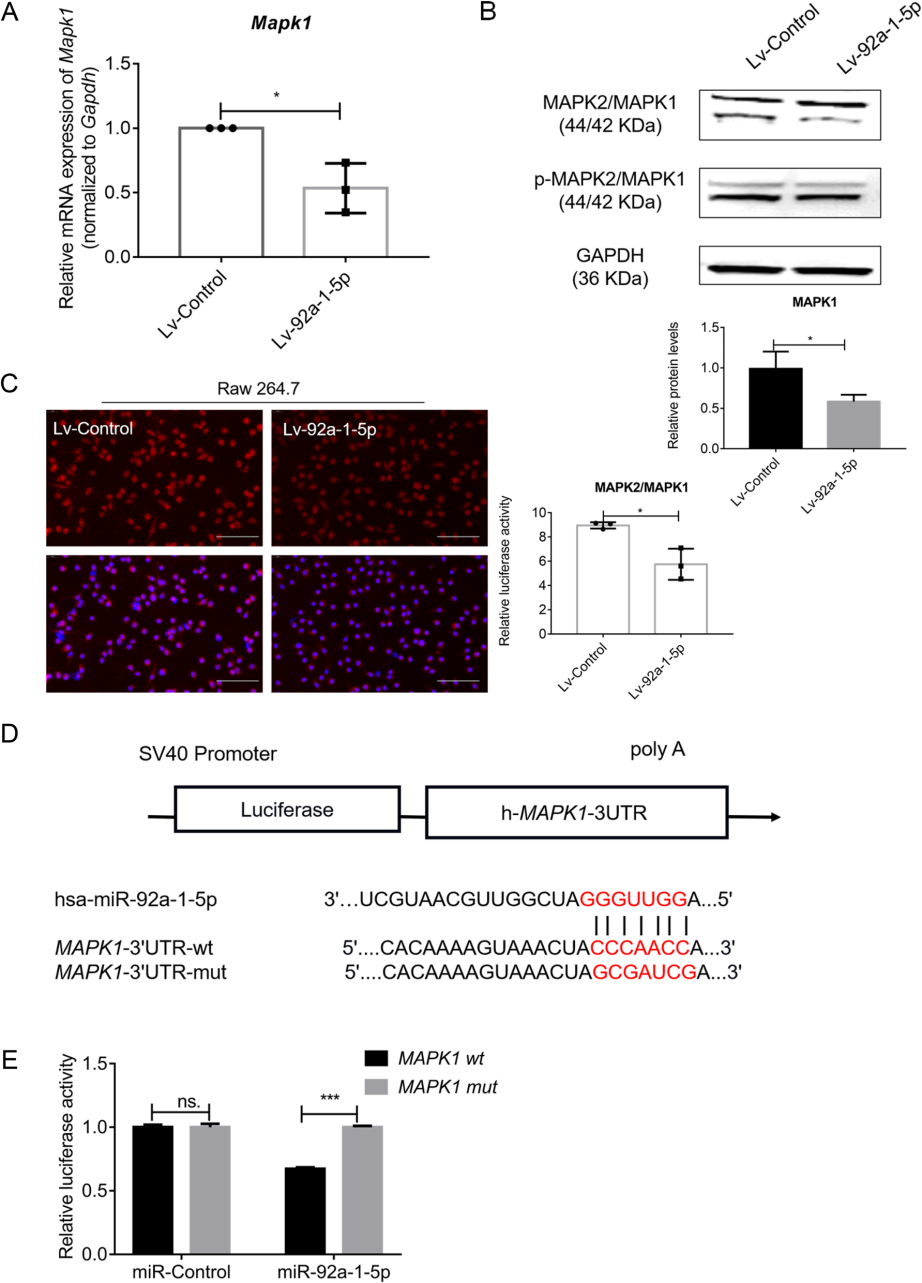
MiR-92a-1-5p directly target MAPK1. **A** *Mapk1* expression determined using qPCR. **B** Western blotting of MAPK1 and pMAPK1 in Raw264.7 cells with stable overexpression of miR-92a-1-5p. GAPDH: internal control. **C** Immunofluorescence labeling of MAPK2/MAPK1 in Raw264.7 cells with stable overexpression of miR-92a-1-5p. The mean fluorescence intensity was decreased. **D** Schematic diagram of the wild and mutant UTRs used. **E** Detection of relative fluorescence intensity at 48 h post co-transfection. Data were analyzed using* t* test. *, *P* < 0.05; ***, *P* < 0.001

To clarify the molecular mechanisms underlying the aforementioned effects, 275 target genes for miR-92a-1-5p were predicted by using miRanda, TargetScan, and miRDB (Fig S4A). We used the tool DIANA to narrow down the targets and analyzed the targets in the top 9 Kyoto Encyclopedia of Genes and Genomes (KEGG) pathways of miR-92a-1-5p. Among these predicted targets, we identified MAPK1 by using the tool DAVID and constructing a PPI network (Fig S4B), in which MAPK1 is one of the most significant modules (green area). Notably, qPCR analysis revealed that MAPK1 mRNA expression in Raw264.7 cells was markedly decreased when miR-92a-1-5p was overexpressed (Fig. 3A), and western blotting results showed that the levels of MAPK1, but not phosphorylated MAPK1 (pMAPK1), were diminished in Lv-92a-1-5p-treated Raw264.7 cells (Fig. 3B). These results were further confirmed by using immunolabeling (Fig. 3C). Through in silico analyses, we found that *MAPK1 3*′-UTR region contains a match site for miR-92a-1-5p (Fig. 3D). Using a dual-luciferase reporter-gene assays, we found that the enzymatic activity was significantly lower in the miR-92a-1-5p co-transfected MAPK1-wt group (Fig. 3E). This result implies that miR-92a-1-5p directly targets *MAPK1*.

### Downregulation of MAPK1 promotes osteoclast differentiation

**Fig. 4:**
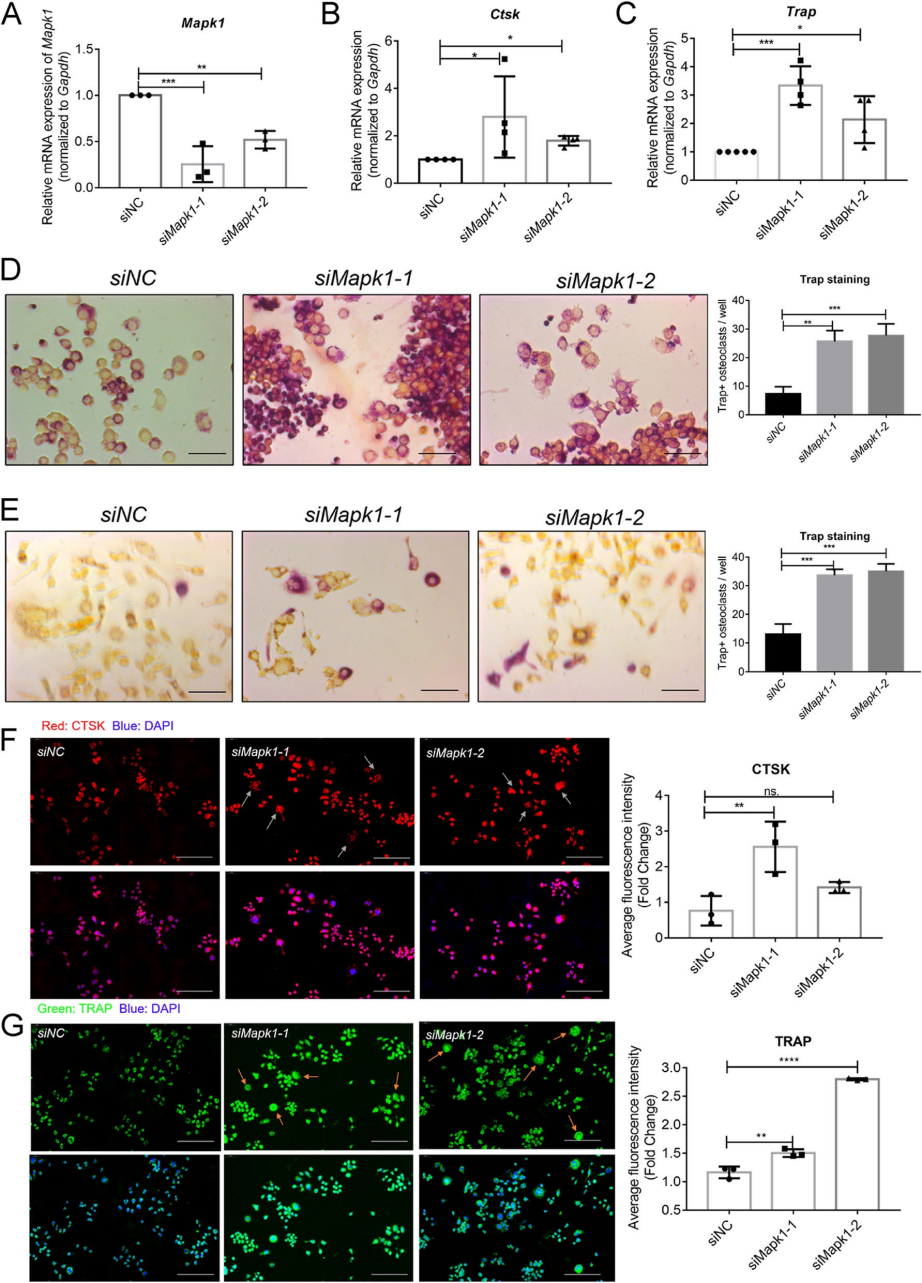
Downregulation of MAPK1 enhances RANKL-mediated osteoclastogenesis. **A** qPCR results confirmed downregulation of *Mapk1* in Raw264.7 cells co-transfected siRNAs. **B-C** *Ctsk* and *Trap* expression determined using qPCR in Raw264.7 cells co-transfected siRNAs in the absence of RANKL. **D-E** Trap + osteoclasts were identified (scale bar: 50 μm) and quantified in Raw264.7 cells (D) and BMMs (E) in the presence of RANKL. The results show an increase of Trap + osteoclasts in the siRNA groups. **F-G** Immunofluorescent staining (scale bar: 100 μm) and quantification of osteoclast markers CTSK and TRAP at 48 h post-transfection in the presence of RANKL. Arrows: multinucleated pre-osteoclasts. Data were analyzed using one-way ANOVA with multiple-comparisons test. *, *P* < 0.05; **, *P* < 0.01; ***, *P* < 0.001

To clarify how MAPK1 downregulation affects osteoclast differentiation, we transfected Mapk1 siRNAs into Raw264.7 cells and using qPCR we confirmed that *Mapk1* expression decreased (Fig. 4A). Notably, transfection of Raw264.7 cells with Mapk1 siRNAs upregulated the Ctsk and Trap mRNA expression (Fig. 4B-C) and increased the Trap + osteoclasts (Fig. 4D-E) in the presence of RANKL. Moreover, increased levels of Ctsk and Trap was confirmed by immunolabeling up to six days post-transfection with Mapk1 siRNAs in the presence of RANKL (Fig. 4F-G).

### Potential role of FoxO1 inhibition in osteoclast differentiation promoted by MAPK1 downregulation

**Fig. 5:**
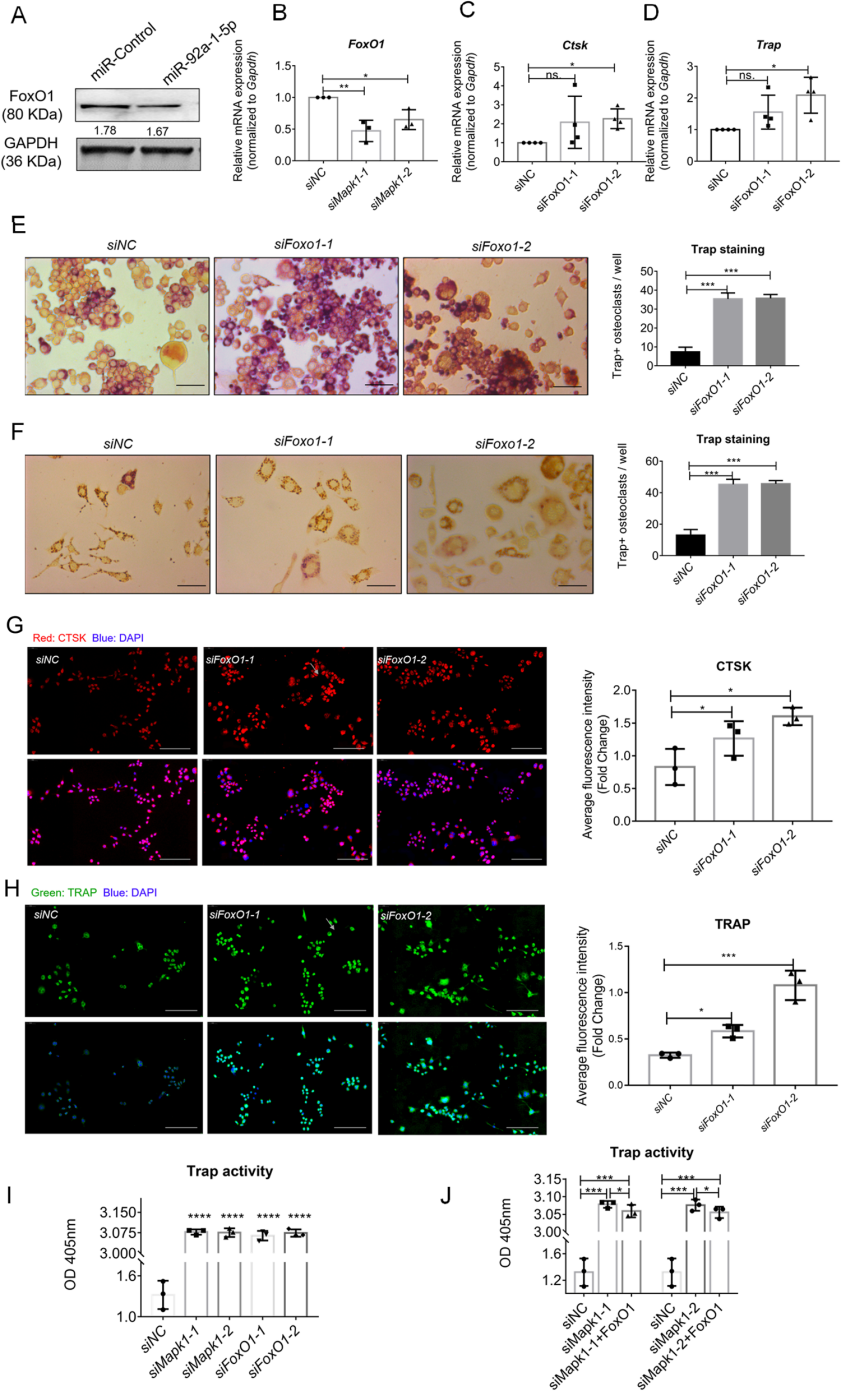
MAPK1 downregulation promotes osteoclast differentiation by decreasing FoxO1 expression. **A** Downregulation of FoxO1 in Raw264.7 cells with stable overexpression of miR-92a-1-5p. GAPDH: internal control. **B** Marked downregulation of FoxO1 mRNA expression following transfection with Mapk1 siRNAs. **C-D** *Ctsk* and *Trap* expression determined by qPCR following transfection with FoxO1 siRNAs. **E–F** Trap + osteoclasts were identified (scale bar: 50 μm) and quantified in Raw264.7 cells (E) and BMMs (F) in the presence of RANKL. **G-H** Immunofluorescent staining (scale bar: 100 μm) and quantification of osteoclast markers CTSK and TRAP at 48 h post-transfection in the presence of RANKL. **I** Trap activity was detected at 6 days post-transfection with indicated siRNAs. **J** Comparison of Trap activity in Raw264.7 cells transfected with indicated siRNAs or siRNAs + FoxO1 fusion protein. Data were analyzed using one-way ANOVA with multiple-comparisons test. *, *P* < 0.05; **, *P* < 0.01; ***, *P* < 0.001

In Raw264.7 cells overexpressing Lv-92a-1-5p, we also unintentionally found FoxO1 downregulation by western blotting (Fig. 5A) and immunolabeling (Fig S5A). However, FoxO1 was not identified as a potential target of miR-92a-1-5p in the bioinformatics analysis performed using TargetScan. Thus, we constructed FoxO1 siRNAs to ascertain whether FoxO1 plays a role in osteoclast differentiation. Whereas FoxO1 mRNA expression was reduced after transfection with Mapk1 siRNAs (Fig. 5B), Mapk1 mRNA expression was unaffected by FoxO1 siRNAs (Fig S5B), which suggested that FoxO1 may be downstream molecular of MAPK1 in osteoclast differentiation. In Raw264.7 cells, transfection with FoxO1 siRNAs upregulated the Ctsk and Trap mRNA expression (Fig. 5C-D), and increased the Trap + osteoclasts (Fig. 5E-F). Furthermore, increased levels of Ctsk and Trap was confirmed by immune-labeling (Fig. 5G-H). The osteoclast differentiation induced by Mapk1 and FoxO1 siRNAs was also confirmed by measuring Trap activity (Fig. 5I).

Next, we investigated whether the osteoclast differentiation mediated by Mapk1 siRNAs depended on FoxO1. After culturing cells for six days, we measured Trap activity in the siRNA NC group, Mapk1 siRNA group, and Mapk1 siRNA + FoxO1 fusion protein group; the results showed that osteoclast differentiation was promoted either in the presence or absence of the FoxO1 fusion protein (Fig. 5J). This finding suggested that although FoxO1 expression was inhibited in response to Mapk1 siRNAs and the downregulation of FoxO1 affected osteoclast differentiation, FoxO1 was not necessary for the osteoclast differentiation induced by Mapk1 siRNAs.

### miR-92a-1-5p enriched EVs promotes osteolysis in vivo

**Fig. 6:**
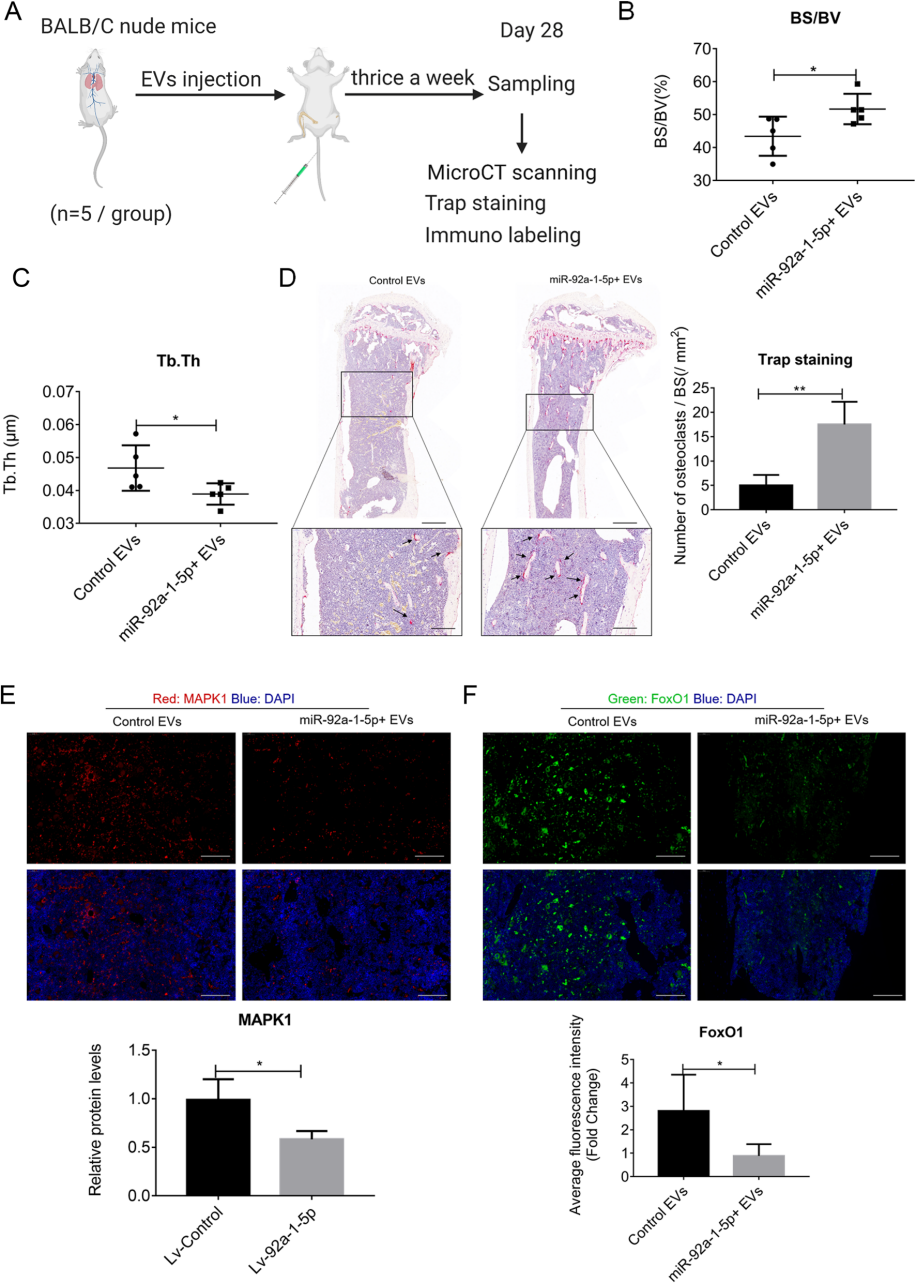
miR-92a-1-5p enriched EVs promotes osteolysis in vivo. **A** Schematic representation of the experiment. **B-C** Micro-CT analysis of BS/TV and Tb.Th in harvested tibia. **D** Representative Trap staining of tibia. The results show an increase in osteoclasts. The arrows show the giant osteoclast cells. Scale bar: 400 μm (upper) and 100 μm (lower). **E–F** Immunofluorescence labeling and quantification of MAPK1 and FoxO1 in tibia. Scale bar: 100 μm. Data were analyzed using *t* test. *, *P* < 0.05; **, *P* < 0.01

Lastly, we investigated the role of miR-92a-1-5p + EVs in vivo. As shown by the results of our previous study [10], bone is the target of PCa EVs. Here, we injected control EVs and miR-92a-1-5p + EVs (10 μg in 100 μL of PBS, thrice weekly) via tail vein for 4 weeks to educate the bone. The results of micro-computed tomography (micro-CT) showed an increased bone surface per bone volume (BS/BV) and decreased trabecular thickness (Tb.Th) in the miR-92a-1-5p + EVs group, both indicating the presence of osteolysis in bone (Fig. 6B-C). However, other measured parameters (bone mineral density (BMD), bone volume per tissue volume (BV/TV), trabecular number (Tb.N), and trabecular separation (Tb.Sp)) did not significantly change (Fig S6). These results suggested a mild role of miR-92a-1-5p + EVs in osteolysis. Furthermore, Trap staining revealed an increase in osteoclasts in the miR-92a-1-5p + EVs group (Fig. 6D), concomitant with a decrease of MAPK1 and FoxO1 (Fig. 6E-F).

### Discussion

We have previously shown that prostate cancer EVs can promote osteoclast differentiation and attenuate osteoblast differentiation, both at least partly mediated by miR-92a-1-5p. In this study, we focused on engineering EVs with potential therapeutic miRNA and investigating their role and cellular mechanisms of action in osteoclast. First, we generate a stable prostate cancer cell overexpressing miR-92a-1-5p resulting in increased miR-92a-1-5p in released EVs. Recipient cells co-cultured with miR-92a-1-5p enriched EVs had a consistent increased expression of miR-92a-1-5p, which is unlikely to be due to recipient cell miR-92a-1-5p production, since pre-miR-92a-1-5p was unaffected by the co-culture with miR-92a-1-5p enriched EVs. These EVs also resulted in increased osteoclast differentiation by increased *ctsk* and *trap* expression in the recipient cells. MiRNA-92a-1-5p directly interacts with MAPK1 mRNA, cause a mutated MAPK1 was not influenced by the miRNA. Further, we mimiced the effects of miR-92a-1-5p enriched EVs by transfecting cells with an siRNA against MAPK1, implying the role of this molecule in miR-92a-1-5p enriched EVs mediated osteoclast differentiation. FoxO1 is considered to be a downstream mediator of MAPK1, and we mimic the miR-92a-1-5p enriched EVs effects with siRNA against this transcription factor. Overall, our results suggest that miR-92a-1-5p enriched EVs mediate osteoclast differentiation via MAPK1 and FoxO1. Repeated exposure of mice with miR-92a-1-5p enriched EVs over four weeks was associated with reduced bone density, and downregulation of MAPK1 and FoxO1 in the bone, suggesting that the pathways we have illustrated in vitro are also relevant in vivo.

When talking about subpopulation of EVs, size heterogeneity is now commonly appreciated [19, 20]. Large EVs (lEVs), small EVs (sEVs) and the newly discovered extracellular particles exomere and supermere have been well investigated in the past years [21-26]. However, one fact is that there is a huge overlap among these subpopulations, not only in size but also in cargoes, which surely limited the further functional study and clinical therapy application of EVs [14, 22]. Based on this, we consider the next step for the EVs subpopulation study should be the isolation of specific cargo positive EVs and clarifying their roles and molecular of actions of these EVs. And in this study, we focused on the subpopulation-miR-92a-1-5p enriched EVs-to clarify their roles and mechanisms.

Since 2007, researchers have discovered that EVs mediate intercellular communication via transferring miRNAs and mRNAs [1] and since then there have been a spurt of growth in the studies of EVs functional roles in intercellular and interorgan communication [4, 27-30]. EVs functional roles are determined by EVs cargoes [19], including but not limited to DNA, RNA, protein and lipids [31, 32]. Among these cargoes, miRNAs have been the most thoroughly investigated [33], small and highly conserved in species [34], which make them as promising EVs therapeutic agents. Nowadays, many studies have revealed the emerging roles of EVs via delivering/encapsuling the endogenous miRNAs [35-37]. However, how to enrich EVs with the exogenous therapeutic miRNAs and use them for disease treatment is still challenging and needed to be addressed before clinical usage. Majority of approaches used for enrichment/loading miRNA into EVs are accomplished by isolating EVs from cell line or body fluids and then loading them with the targeted miRNA by using chemical or physical methods [11]. However, contamination of reagent, aggregation of EVs or miRNA, and leakage of EVs cargoes during the process, have been drawing increasing attention and may cause safety issue. Here, we proposed an assay to produce EVs carrying the desired therapeutic miRNA as a valid alternative to the previously described methods.

In the choice of this original cells for the designed EVs, we choose MDA PCa 2b cell line. We previously reported that EVs derived from osteoblastic MDA PCa 2b cells promote osteoclast differentiation [10] and here we aim to strengthen the osteolysis functions of MDA PCa 2b EVs by overexpressing miR-92a-1-5p. After four weeks bone marrow education, miRNA-92a-1-5p enriched EVs promote osteolysis with a change in bone surface per bone volume (BS/BV) and decreased trabecular thickness (Tb.Th) (Fig. 6B-C) and no change in other measured parameters (bone mineral density (BMD), bone volume per tissue volume (BV/TV), trabecular number (Tb.N), and trabecular separation (Tb.Sp)) (Figure S5), which indicates a mild osteolysis process, resulting as a potential safety approach for further therapy application.

We also found that miR-92a-1-5p enriched EVs work as positive regulator of osteoblast differentiation in both Raw264.7 cells and BMMs, which are two most commonly used systems for osteoclast differentiation [38]. Moreover, in vivo osteolysis induced by miR-92a-1-5p enriched EVs was also confirmed after four weeks bone marrow education via tail injection. Although only with a two fold upregulation of miR-92a-1-5p in miR-92a-1-5p enriched EVs compared to control EVs, evidence of osteolysis is obvious, indicating the high-efficiency of the engineered EVs. A plausible explanation for this high efficiency is the involvement of multiple targets of miR-92a-1-5p. We previously clarified miR-92a-1-5p promote osteoclast differentiation and concurrently inhibit osteogenesis by targeting COL1A1 [10]. Previous study already identified a link between MAPK1, Dock10 and FoxO1 as a key signalling axis that incorporates MAPK1 signalling to promote cancer progression through induction of EMT, increased invasiveness and resistance to compression [39]. In another study, based on that FoxO1 contains 15 consensus phosphorylation sites for the mitogen-activated protein kinase (MAPK) family, researchers confirmed that MAPK1 could directly regulate the transcriptional activity of FoxO1 via phosphorylation [40]. Here, we confirmed MAPK1 as the target of miR-92a-1-5p to promote osteoclast differentiation, meanwhile downregulation of Mapk1 also induced decrease of FoxO1, leading to further osteoclast differentiation. Trap activity was restored with the adding of FoxO1 fusion protein, indicating that downregulation of FoxO1 at least partly contribute to the osteoclast differentiation mediated by MAPK1 downregulation. We demonstrated that miR-92a-1-5p promotes osteoclast differentiation via targeting Mapk1 in a FoxO1 independent manner. However, how much FoxO1 downregulation played during the process still requires further investigation. Until now, up to our knowledge, there has been no evidence for direct binding of MAPK1 and FoxO1.miR-92a-1-5p was identified only recently and still only few studies have been reported [41-43]. There are more than 200 predicted targets for miR-92a-1-5p in the intersection of the three miRNA target gene databases, some of which are very relevant in the process of osteoclast differentiation (e.g., COL1A1, BMP7, MMP2, TGFBR3, IL10, COL27A1 and FOXF1). The involvement of other potential targets should be elucidated in future studies.

Interestingly, miRNA-92a-1-5p was upregulated in MDA PCa 2b EVs, however, the circulating exosomal miRNA-92a-1-5p was downregulated in patient serum (Figure S7). We noticed that deep sequencing results from other researchers suggested exosomal miR-92a-1-5p downregulation in urine exosomes of PCa patients [44]. Further, we detected the tissue exosomal miR-92a-1-5p expression in PCa mice models and, as expected, it was upregulated in prostate comparison with non-tumor group (data not shown). Based on these evidences, one possible explanation for this discrepancy is that exosomal miRNA-92a-1-5p may be preserved in PCa cell and tissue derived EVs to functional their roles. Meanwhile, the clearance of miR-92a-1-5p was decreased via lower expression in serum and urine EVs. This explanation is in agreement with the results of other research: The protein compositions of PDX mice tissue derived exosomes and human cell line derived exosomes were well correlated. However, the consistency between human tumor cell derived exosomes and serum exosome cargos was poor [45].

### Conclusion

Collectively, here we reported a miRNA enriched EVs constructing system to construct the miR-92a-1-5p enriched PCa EVs subpopulation. Further we clarified the roles and mechanisms of this designed EVs in osteolysis: miR-92a-1-5p enriched PCa EVs mediate osteoclast differentiation via MAPK1 and FoxO1, which may benefit the therapy of osteoblastic bone disease in the future.

## Supplementary Information

**Additional file 1:** **Figure S1. **Diagram of lentiviral construct for miR-92a-1-5p overexpression. **Figure S2.** Characterization of miR-92a-1-5p+ EVs. A Representative images of EVs in TEM. Scale bar = 200 nm. B Representative size distribution of the EVs, measured by NTA, with the main peak appearing at 90–130 nm. C Western blotting of GM130, Alix, CD9, and Annexin V from 10 μg of EVs. D Flow cytometry analysis of CD63 and CD81 in the designed EVs and Control EVs. E EVs yield was determined by BCA. **Figure S3. **TRAP and CTSK expression after 48 h EVs co-culture with Raw264.7 cells. A Western blotting result; B-C Gray analysis of western blotting. Data were analyzed using *t* test. *, *P* < 0.05, **, *P* < 0.01. **Figure S4.** MiR-92a-1-5p directly target MAPK1. A Bioinformatic prediction of miR-92a-1-5p target genes. B PPI network of functional targets, constructed using STRING. The most significant module is shown in green. **Figure S5. **MAPK1 downregulation promoted osteoclast differentiation by decreasing FoxO1 expression. A Immunofluorescencestaining (left) and luciferase-reporter assay results (right) showing FoxO1 downregulation in Lv-92a-1-5p group. B *Mapk1* mRNA expression was unaffected by FoxO1 siRNAs. Data were analyzed using *t* test (A) and one-wayANOVA with multiple-comparisons test (B). **, *P* < 0.01; ns, not significant. **Figure S6.** Micro-CT analysis after 4 weeks of bone marrow education. The parameters BMD, BVF,Tb.N, and Tb.Sp did not change significantly. Data were analyzed using t test. **Figure S7.** Circulating exosomal miR-92a-1-5p may serve as biomarker for bone metastatic PCa. A Representative images of serum EVs in TEM. Scale bar = 200 nm. B Flow cytometry analysis of CD63 and CD81 in serum EVs. C qPCR analysis of relative expression levels of serum exosomal miR-92a-1-5p in BPH group (*n* = 12) and PCa group (*n* = 35). The results show drastic downregulation of exosomal miR-92a-1-5p in PCa group. D qPCR analysis of relative expression levels of serum exosomal miR-92a-1-5p in BPH group (*n* = 12), localized PCa group (*n* = 11), and bone metastatic PCa group (*n* = 12). The results show downregulation of exosomal miR-92a-1-5p in localized PCa group and bone metastatic PCa group. E Analysis of correlation between exosomal miR-92a-1-5p and serum ALP in PCa patients (*n* = 23). F Analysis of correlation between exosomal miR-92a-1-5p and serum ALP in bone metastatic PCa patients (*n* = 12). G Analysis of correlation between tPSA and serum ALP in bone metastatic PCa patients (*n* = 12). H Analysis of correlation between fPSA and serum ALP in bone metastatic PCa patients (*n* =12). I ROC curve of exosomal miR-92a-1-5p to distinguish BPH and bone metastatic PCa. Data were analyzed using t test (c), one-way ANOVA with multiple-comparisons test (d), linear regression analysis (e-h) and ROC analysis (i). *, *P* < 0.05; **, *P*< 0.01; ***, *P* < 0.001. **Table S1.** Primer sequencesused for qPCR.

## Data Availability

All the original data are available upon reasonable request for correspondence authors.

## Abbreviations

BMMs: Bone marrow macrophages
EVs: Extracellular vesicles
EMT: Epithelial-mesenchymal transition
FBS: Fetal bovine serum
KEGG: Kyoto Encyclopedia of Genes and Genomes
lEVs: Large EVs
miRNAs: MicroRNAs
MOI: Multiplicity of infection
NC: Negative Control
NTA: Nanoparticle tracking analysis
PBS: Phosphate-buffered saline
PCa: Prostate cancer
sEVs: Small EVs
TEM: Transmission electron microscopy
