# Supplementary material for: miR-92a-1-5p enriched prostate cancer extracellular vesicles regulate osteoclast function via MAPK1 and FoxO1
Source: J Exp Clin Cancer Res. 2023 May 2;42:109. doi: 10.1186/s13046-023-02685-2 (PMC10152631; doi:10.1186/s13046-023-02685-2)
Supplement: Supplementary file 1 — Additional file 1: Figure S1. Diagram of lentiviral construct for miR-92a-1-5p overexpression. Figure S2. Characterization of miR-92a-1-5p+ EVs. A Representative images of EVs in TEM. Scale bar = 200 nm. B Representative size distribution of the EVs, measured by NTA, with the main peak appearing at 90–130 nm. C Western blotting of GM130, Alix, CD9, and Annexin V from 10 μg of EVs. D Flow cytometry analysis of CD63 and CD81 in the designed EVs and Control EVs. E EVs yield was determined by BCA. Figure S3. TRAP and CTSK expression after 48 h EVs co-culture with Raw264.7 cells. A Western blotting result; B-C Gray analysis of western blotting. Data were analyzed using t test. *, P < 0.05, **, P < 0.01. Figure S4. MiR-92a-1-5p directly target MAPK1. A Bioinformatic prediction of miR-92a-1-5p target genes. B PPI network of functional targets, constructed using STRING. The most significant module is shown in green. Figure S5. MAPK1 downregulation promoted osteoclast differentiation by decreasing FoxO1 expression. A Immunofluorescencestaining (left) and luciferase-reporter assay results (right) showing FoxO1 downregulation in Lv-92a-1-5p group. B Mapk1 mRNA expression was unaffected by FoxO1 siRNAs. Data were analyzed using t test (A) and one-wayANOVA with multiple-comparisons test (B). **, P < 0.01; ns, not significant. Figure S6. Micro-CT analysis after 4 weeks of bone marrow education. The parameters BMD, BVF,Tb.N, and Tb.Sp did not change significantly. Data were analyzed using t test. Figure S7. Circulating exosomal miR-92a-1-5p may serve as biomarker for bone metastatic PCa. A Representative images of serum EVs in TEM. Scale bar = 200 nm. B Flow cytometry analysis of CD63 and CD81 in serum EVs. C qPCR analysis of relative expression levels of serum exosomal miR-92a-1-5p in BPH group (n = 12) and PCa group (n = 35). The results show drastic downregulation of exosomal miR-92a-1-5p in PCa group. D qPCR analysis of relative expression levels of serum exosomal miR-92a-1-5p [file 13046_2023_2685_MOESM1_ESM.docx]

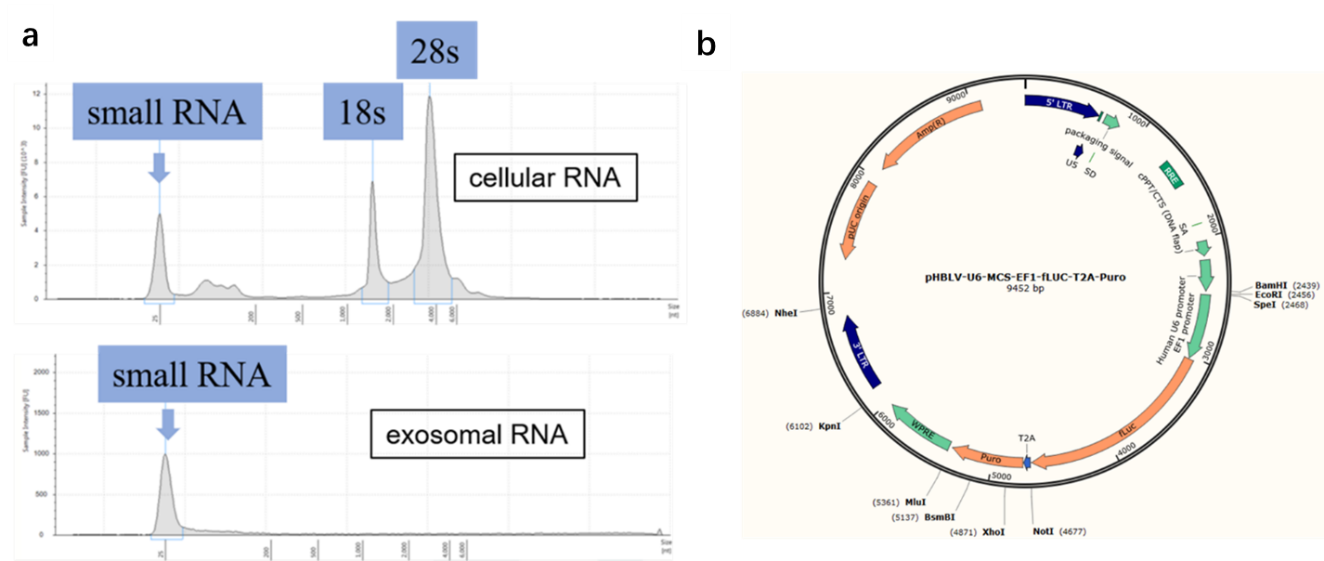


Figure S1. Diagram of lentiviral construct for miR-92a-1-5p overexpression.


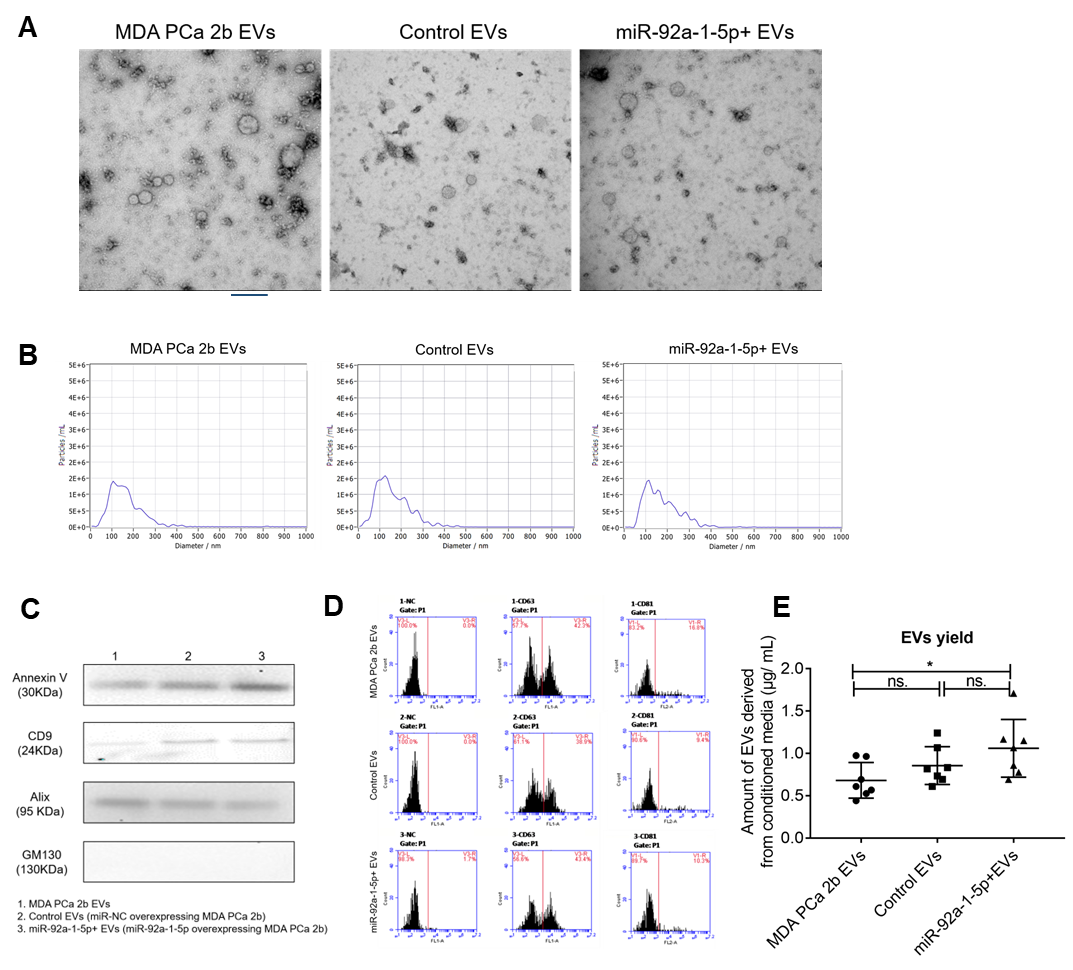


**Figure S2. Characterization of miR-92a-1-5p+ EVs.** A Representative images of EVs in TEM. Scale bar = 200 nm. B Representative size distribution of the EVs, measured by NTA, with the main peak appearing at 90–130 nm. C Western blotting of GM130, Alix, CD9, and Annexin V from 10 μg of EVs. D Flow cytometry analysis of CD63 and CD81 in the designed EVs and Control EVs. E EVs yield was determined by BCA.


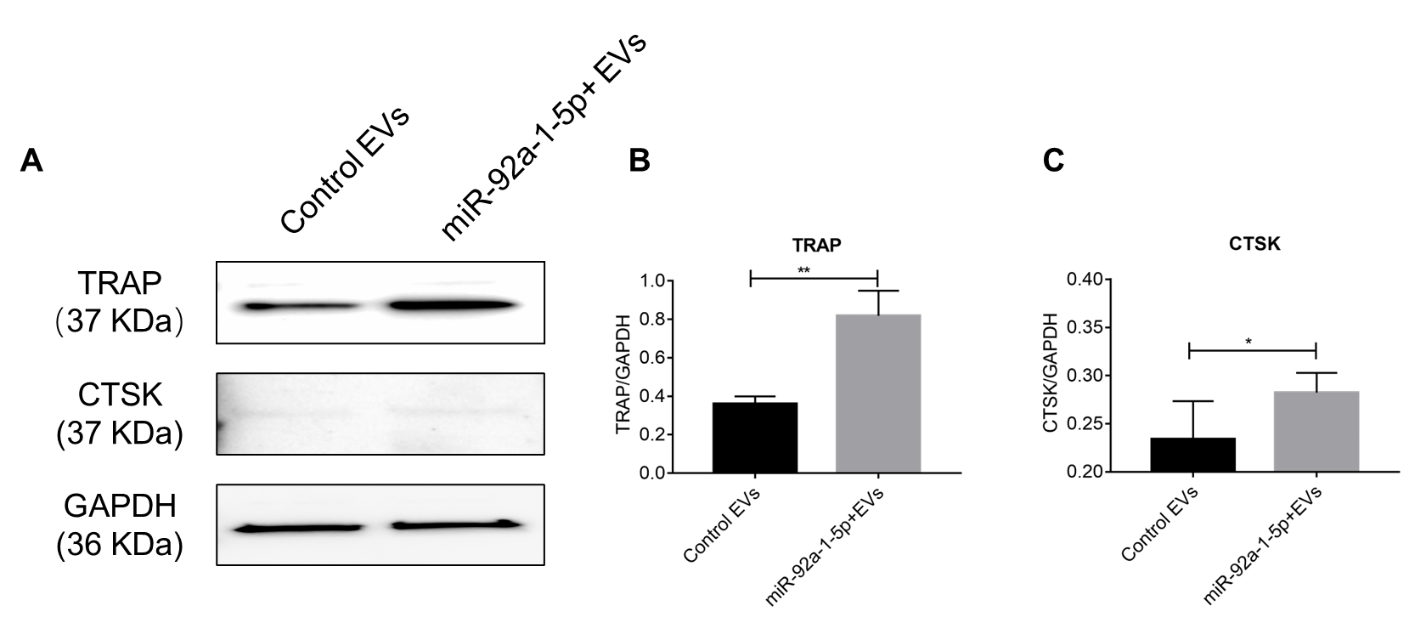


**Figure S3. TRAP and CTSK expression after 48 h EVs co-culture with Raw264.7 cells.** **A** Western blotting result; **B-C** Gray analysis of western blotting. Data were analyzed using *t* test. *, *P* < 0.05, **, *P* < 0.01.


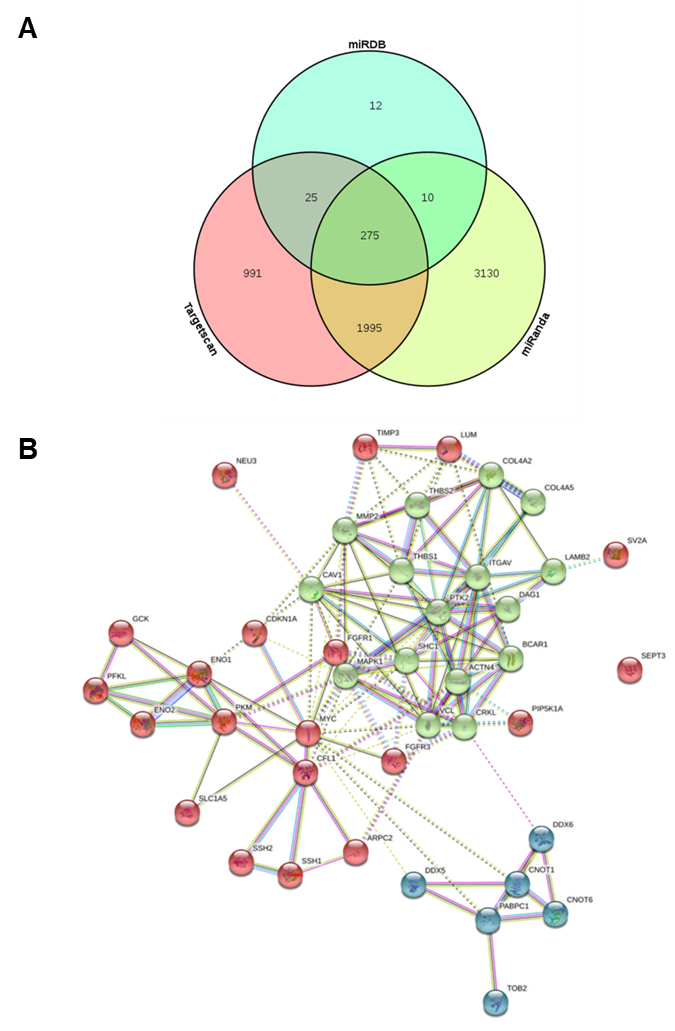


Figure S4. MiR-92a-1-5p directly target MAPK1. A Bioinformatic prediction of miR-92a-1-5p target genes. B PPI network of functional targets, constructed using STRING. The most significant module is shown in green.


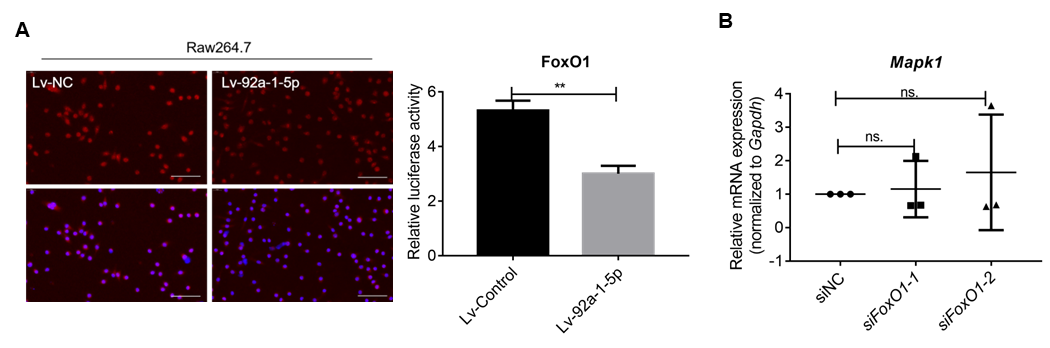


Figure S5. MAPK1 downregulation promoted osteoclast differentiation by decreasing FoxO1 expression. A Immunofluorescence staining (left) and luciferase-reporter assay results (right) showing FoxO1 downregulation in Lv-92a-1-5p group. B *Mapk1* mRNA expression was unaffected by FoxO1 siRNAs. Data were analyzed using *t* test (A) and one-way ANOVA with multiple-comparisons test (B). **, *P* < 0.01; ns, not significant.


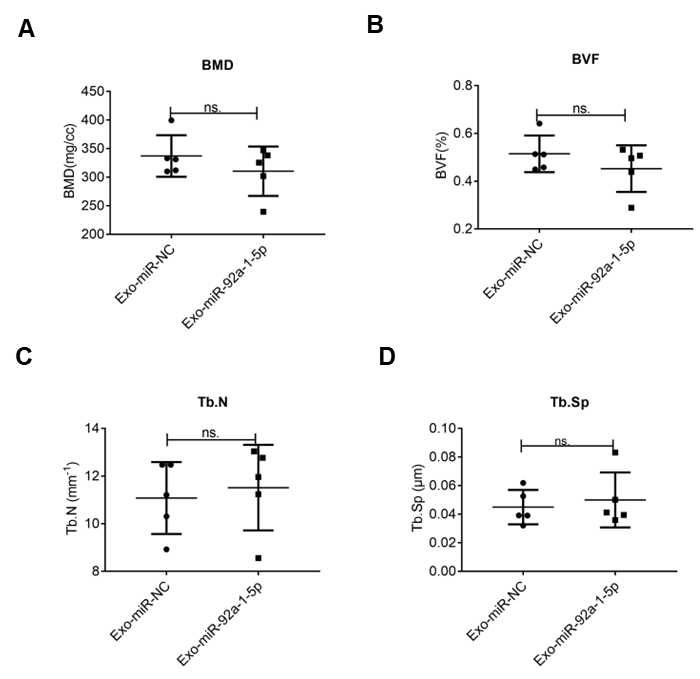


Figure S6. Micro-CT analysis after 4 weeks of bone marrow education. The parameters BMD, BVF, Tb.N, and Tb.Sp did not change significantly. Data were analyzed using t test.


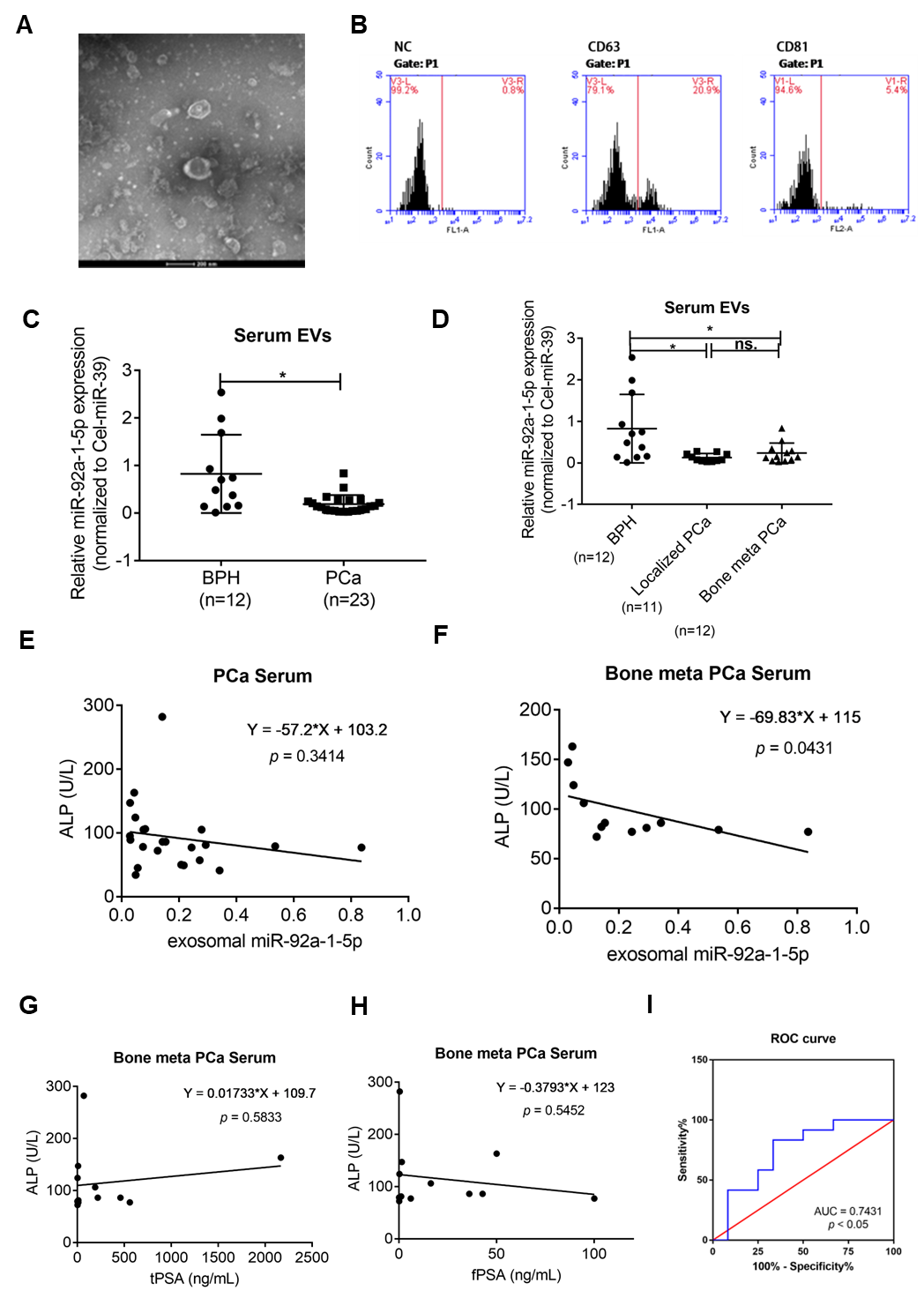


Figure S7. Circulating exosomal miR-92a-1-5p may serve as biomarker for bone metastatic PCa. A Representative images of serum EVs in TEM. Scale bar = 200 nm. B Flow cytometry analysis of CD63 and CD81 in serum EVs. C qPCR analysis of relative expression levels of serum exosomal miR-92a-1-5p in BPH group (n = 12) and PCa group (n = 35). The results show drastic downregulation of exosomal miR-92a-1-5p in PCa group. D qPCR analysis of relative expression levels of serum exosomal miR-92a-1-5p in BPH group (n = 12), localized PCa group (n = 11), and bone metastatic PCa group (n = 12). The results show downregulation of exosomal miR-92a-1-5p in localized PCa group and bone metastatic PCa group. E Analysis of correlation between exosomal miR-92a-1-5p and serum ALP in PCa patients (n = 23). F Analysis of correlation between exosomal miR-92a-1-5p and serum ALP in bone metastatic PCa patients (n = 12). G Analysis of correlation between tPSA and serum ALP in bone metastatic PCa patients (n = 12). H Analysis of correlation between fPSA and serum ALP in bone metastatic PCa patients (n = 12). I ROC curve of exosomal miR-92a-1-5p to distinguish BPH and bone metastatic PCa. Data were analyzed using t test (c), one-way ANOVA with multiple-comparisons test (d), linear regression analysis (e-h) and ROC analysis (i). *, *P* < 0.05; **, *P* < 0.01; ***, *P* < 0.001.

Table S1.

Primer sequences used for qPCR.

| Gene | Forward (5ʹ-3ʹ) | Reverse (5ʹ-3ʹ) |
| --- | --- | --- |
| *Gapdh* | GGTGAAGGTCGGTGTGTGAACG | CTCGCTCCTGGAAGATGGTG |
| *Ctsk* | GGCCAGTGTGGTTCCTGTTGG | CCGCCTCCACAGCCATAATTCTC |
| *Trap* | CACTCCCACCCTGAGATTTGT | CATCGTCTGCACGGTTCTG |
| *Mapk1* | TCTCCTCTGTGTTGTCCTCCTTCC | GGCTGCCGCTCGACTTATGC |
| *FoxO1* | ACATCTGCCATGAACCGCTTGAC | CACCCATCCTACCATAGCCATTGC |
